# Intracellular Trafficking Network and Autophagy of PHBHHx Nanoparticles and their Implications for Drug Delivery

**DOI:** 10.1038/s41598-019-45632-y

**Published:** 2019-07-03

**Authors:** Xiangyu Sun, Cheng Cheng, Jinxie Zhang, Xing Jin, Shuqing Sun, Lin Mei, Laiqiang Huang

**Affiliations:** 1 Graduate School at Shenzhen, Tsinghua University, Shenzhen, 518055 China; 2 Department of Physics, Tsinghua University, Beijing, 100084 China; 3 College of chemistry and pharmaceutical engineering, Jilin Institute of Chemical Technology, Jilin, 132022 China; 4 School of Life Sciences, Tsinghua University, Beijing, 100084 China; 5 School of Pharmaceutical Sciences (Shenzhen), Sun Yat-sen University, Guangzhou, 510275 China

**Keywords:** Drug delivery, Drug delivery

## Abstract

3-hydroxybutyrate-co-3-hydroxyhexanoate (PHBHHx), which is naturally generated by biodegradable polyhydroxyalkanoates synthesized by bacteria, is an attractive material for drug delivery due to its controllable physical properties, non-toxicity, environmental friendliness, degradable properties and good biocompatibility. However, the intracellular trafficking network pathways, especially the autophagy mechanism of PHBHHx nanoparticles (NPs), have rarely been investigated. In this paper, we successfully prepared the NPs used solvent displacement method and investigated the autophagy pathways and other intracellular trafficking mechanisms based on NPs with the assistance of Rab proteins. We found that NPs were internalized in cells mainly via clathrin endocytosis and caveolin endocytosis. Beside the classical pathways, we discovered two new pathways: the micropinocytosis early endosome (EEs)-micropinocytosis-lysosome pathway and the EEs-liposome-lysosome pathway. NPs were delivered to cells through endocytosis recycling vesicles and GLUT4 exocytosis vesicles. Similar to other nanoparticles, NPs also induced intracellular autophagy and were then degraded via endolysosomal pathways. 3-MA and CQ were used as autophagy inhibitors to avoid the degradation of NPs through lysosomes by blocking endolysosomal pathways. Tumor volumes and weights were significantly decreased when autophagy inhibitors and chemical drugs packaged in NPs were cooperatively used.

## Introduction

Application of nanoparticles to enhance the effectiveness of diagnostic agents and drugs is becoming a part of our medical armament^1–4^. More attention should be paid to novel nanoparticles or the diagnosis and treatment of different diseases^5–8^. Nanoparticles are used as tools to encapsulate and deliver traditional chemotherapeutic agents^9^. To enhance the therapeutic effects of traditional drugs, drug carriers are expected to have connections with the exterior of membranes to help deliver drugs to the cellular environmental^10^. Nano-sized features offer certain advantageous properties for nanoparticles on this journey and the intracellular uptake is the important step of the journey for nanoparticles to reach the bio-active site^11^. Obviously, in-depth understanding of the intracellular transport pathways is critical for the advancement of studies on nanotechnology and drug delivery.

3-hydroxybutyrate- co-3-hydroxyhexanoate (PHBHHx) is a copolymer naturally generated by biodegradable polyhydroxyalkanoates, which are synthesized by bacteria^12^. PHBHHx copolymer can dissolved into the common organic solvent such as methylene dichloride which make it a good candidate to apply to the biology and chemistry research field. Because it comes from microbes, PHBHHx NPs has unique characteristics due to its controllable physical and chemical properties, environmental friendliness, good degradable properties and biocompatibility when compared to other nanocarrier particles, including carbon nanotubes, liposome micelles, silica nanoparticles, dendrimers, solid lipid nanoparticles, anodic alumina nanotubes^13–18^. However, little research on the intracellular trafficking pathways of PHBHHx nanostructured materials has been made. In this article, we report a comprehensive investigation on the intracellular trafficking pathway of PHBHHx nanoparticles (NPs). 3-MA and CQ were used as autophagy inhibitors to avoid the degradation of PHBHHx NPs, and their implications for drug delivery are also explored. The volumes and weights of the tumors were significantly decreased when autophagy inhibitors and chemical drugs packaged in PHBHHx NPs were cooperatively used. Biological studies can provide supportive information on the understanding of other spherical nanostructured materials as well.

Rab GTPases are a family of proteins that act as coordinators of the vesicular trafficking pathways that are responsible for transporting the vast array of cellular cargo across membrane organelles^19^. Approximately 70 human Rab proteins have been discovered, most of which show close relationships with the trafficking of vesicles in and across intracellular trafficking vesica^20^. In this paper, Rab was used as a marker to explore the intracellular trafficking mechanism of PHBHHx NPs.

## Results and Discussion

### Preparation and characterization of PHBHHx NPs

**Figure 1:**
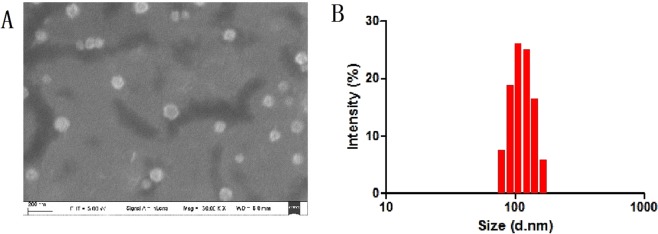
SEM characterization of PHBHHx NPs (**A**) DLS size distribution of PHBHHx NPs (**B**).

As shown in the SEM images in Fig. 1, the size of the PHBHHx NPs was approximately 110 nm, the size distribution analysis by DLS measurement (Fig. 1B) also shows good agreement with the SEM results. The zeta potential of the NPs was measured to be −10 ± 0.3 mV, indicating the negatively charged nature of the materials. The LC and EE of the paclitaxel-NPs were 7.41% and 68.36%, respectively.

### Endocytosis pathways of PHBHHx NPs

The endocytosis pathways are characterized by the engulfment of extracellular macromolecules, such as proteins, other foreign invader-like nanoparticles, or membrane constituents, via membrane invagination^21^. There are two classical endocytosis pathways: clathrin -dependent and clathrin-independent. Clathrin-dependent endocytosis can be subdivided into caveolin-dependent, caveolin-independent and macropinocytosis pathways based on the involvement of membrane proteins called caveolins. The caveolin-independent pathway can be further divided into Arf-6, RhoA-dependent, Cdc42 and flotillin pathways^22^. The classical endocytosis pathways of nanoparticles begin with their sequestration in vesicles, and then their delivery to EEs, LEs, and subsequently lysosomes as the final destination^23^.

**Figure 2:**
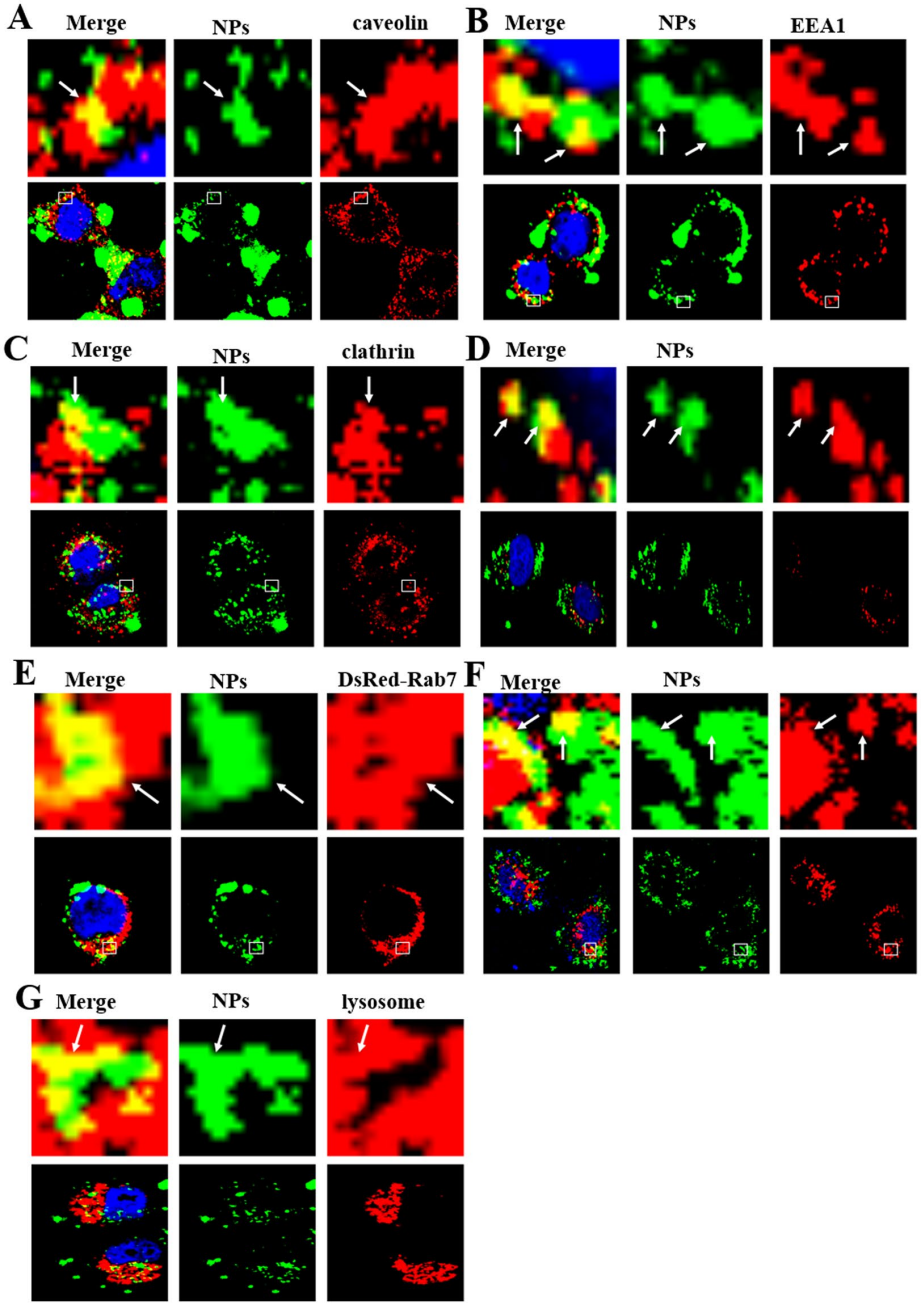
Confocal images of endocytosis pathways. In MCF-7 cells, the NPs enter the cells through clathrin-dependent and caveolin-dependent endocytosis (**A**–**C**) after treatment with 1 mg/mL coumarin-6-labeled NPs for 3 h. Clathrin, caveolin and EEA1 were detected by primary antibodies against clathrin, caveolin and EEA1. (**D**–**F**) DsRed-Rab5, 7 and 9 cells were incubated with 1 mg/mL coumarin-6-labeled NPs for 3 h. (**F**) 1 mg/mL coumarin-6-labeled NPs transfected MCF-7 cells for 3 h and detected by Lyso-Tracker Red probes for 30 min. The above images are the enlarged ones in the white collar on the underside images. Scale bars: 10 μm.

Coumarin-6-labeled NPs were prepared to identify the specific internalization pathways. MCF-7 cells were cultured with 1 mg/mL Coumarin-6-labeled NPs at 37 °C for 3 h, and the Coumarin-6-positive vesicles co-localized with caveolin-positive vesicles and clathrin-dependent positive vesicles (Figs 2A, S1A–D), rather than with other endocytosis-related vesicles (i.e., Arf-6, clathrin, flotillin, Cdc42, and RhoA-positive vesicles). This result clearly shows that cells internalize PHBHHx nanoparticles through clathrin-dependent pathways and caveolin-dependent endocytosis, a subtype of clathrin-independent pathway (Fig. 2A–C).

A large array of Rab proteins has been identified, and they were confirmed to have a close relationship with vesicle trafficking and transport^24^. To validate the detailed endocytosis pathways of NPs, we use labeled Rab family proteins as markers. The first experiment carried out was a study on the relationship between NPs and two organelles (EEs and LEs) in classic endocytosis pathways using DsRed-Rab5, 7 and 9 proteins as probes.

The cells transfected by DsRed-Rab5, 7, and 9 were incubated with coumarin-6-labeled NPs at 37 °C for 3 h. As shown in Fig. 2B,D, Coumarin-6-containing vesicles merged with EEA1 and DsRed-Rab5, the markers of early endosomes. Meanwhile, coumarin-6-positive vesicles also fused with DsRed-Rab7 and DsRed-Rab9, the markers of late endosomes (Fig. 2E,F). These images revealed a classical endocytosis pathway involved in the process of NP endocytosis. This postulation can be further demonstrated by the observation of merged images of Coumarin-6-positive vesicles and lysosomes (Fig. 2G). These experiments provided an inside view of NP endocytosis pathways, indicating that the NPs were transported to EEs and LEs and degraded in lysosomes. This finding is identical to the classical endocytosis pathways.

**Figure 3:**
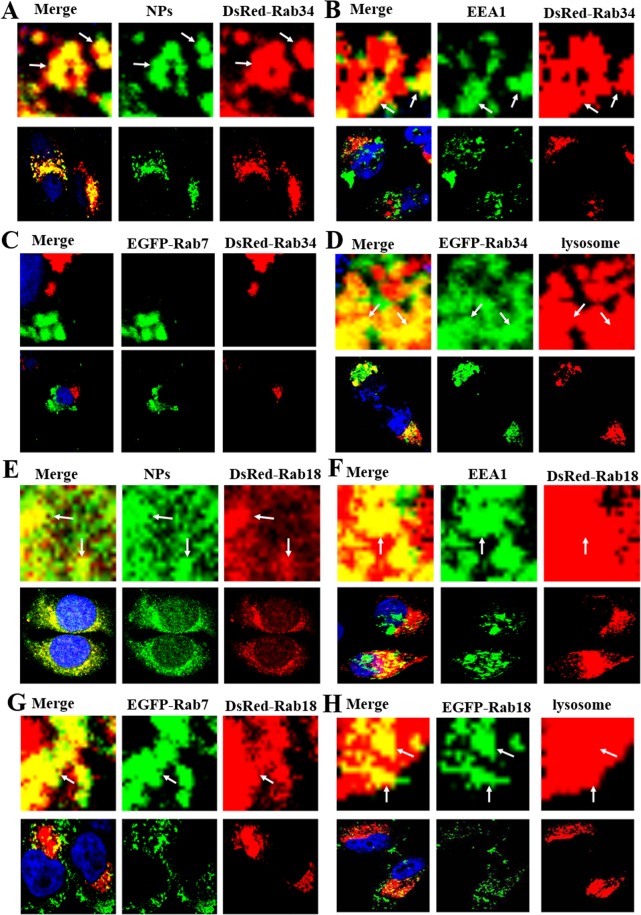
Confocal microscopy pictures of endocytosis pathways. (**A**) MCF-7 cells were transfected by DsRed-Rab34 and then cultured with 1 mg/mL coumarin-6-labeled NPs for 3 h. (**B**) DsRed-Rab34 cells were cultured with 1 mg/mL NPs for 3 h; EEA1 was detected with a primary antibody against EEA1. (**C**) DsRed-Rab34 cells were co-cultured with EGFP-Rab7 and 1 mg/mL NPs for 3 h. (**D**) EGFP-Rab34 cells were cultured with 1 mg/mL NPs for 3 h and Lyso-Tracker Red probes for 30 min. (**E**–**H**) The same procedure was repeated, except that DsRed-Rab34 was replaced by DsRed-Rab18. The above images are the enlarged ones in the white collar on the underside images. Scale bars: 10 μm.

In addition to studies on EEs and LEs, other Rab proteins were used to analyze other pathways. Among Rab proteins, Rab18 is highly associated with the activity of lipid droplets, and Rab34 is a biomarker of the micropinocytosis process; Rab18 and Rab34 can therefore be used to illustrate the roles of lipid droplets and micropinocytosis, respectively, in NP endocytosis pathways^25,26^. DsRed-Rab18 and DsRed-Rab34 transfected MCF-7 cells were cultured at 37 °C for 3 h. Both Rab18 and Rab34 were observed to merge with NP-containing vesicles (Fig. 3A,E). To obtain more detailed information on the role of lipid droplets in the endocytosis pathways, the reactions between lipid droplets and markers for classical endocytosis paths (EEA1 and EGFP-Rab7) were also examined. Figure 3F–H shows the integration of Rab18-positive vesicles with EEA1, lysosomes, and EGFP-Rab7. This finding serves as proof of the participation of liposomes in the endocytosis pathways of NPs. This finding was also applied to studies on DsRed-Rab34, EGFP-Rab7 and lysosomes in the micropinocytosis process. To our surprise, Rab34 was found to merge with EEA1, lysosome but not EGFP-Rab7 (Fig. 3B–D). All this evidence points to the existence of a new endocytosis pathway: EEs (EEA1-positive)-macropinocytosis (Rab34 -positive)-lysosomes.

### Recycling endosome pathways of NPs

The recycling endosome is an important organelle for the redelivery of protein receptors back to cell membranes from EEs^27^. According to previous reports, the action of recycling endosomes is facilitated by numerous accessory proteins. DsRed-Rab11 and DsRed-Rab35 participate in the process of slow endocytic recycling, and DsRed-Rab20 and DsRed-Rab25 are highly engaged in transportation between the apical recycling endosomes and apical plasma membrane^28^.

Herein, we report a study on intracellular trafficking pathways. MCF-7 cells were transfected with DsRed-Rab 11, 20, 25 and 35 and incubated with Coumarin-6-labeled NPs at 37 °C for 3 h. As shown in Figure S2A,B Rab11 and Rab35 marked slow recycling endosomes that fused with Coumarin -6-positive NPs, which also occurred on recycling endosomes marked with Rab20 and Rab25 (Figure S2C,D). As shown above, both slow and apical recycling endosome paths participate in the process of releasing NPs back to the cell membrane. The metabolic pathways of NPs started from clathrin -dependent pathways and caveolin-dependent endocytosis. Through these two processes, NPs were internalized in the cell and further transported to EEs, LEs, and lysosomes in classic endocytosis pathways. In addition, a new chain with the participation of EEs, macropinocytosis and lysosomes was discovered. Then, at the late stage of this metabolic pathway, both slow and apical recycling endosomes were observed in the inside-out process of NPs. Rab18-labeled liposomes were also observed in classical endocytosis pathways.

### Exocytosis pathways of NPs

Secretory vesicles release their contents out of the cell through exocytosis^29^. This action is assisted by a group of Rab proteins. Recent studies on NPs showed that DsRed-Rab8, 10, and 14 regulate the flow of GLUT4 vesicles on Golgi^20^. To determine whether NPs can be transported out of the cell through pathways that the labeled proteins could monitor, DsRed-Rab8 and DsRed-Rab10 transfected MCF-7 cells were used. The transfected cells were further incubated with Coumarin -6-labeled NPs at 37 °C for 3 h. Figure S3A,B presents a merged image of Rab-positive vesicles (GLUT4 vesicle, marked with DsRed-Rab8 and Rab10) and Coumarin -6-positive vesicles. This evidence points to the involvement of Rab8- and Rab10-positive GLUT4 vesicle in the exocytosis process of NPs. Two critical procedures occur in the GLUT4-related pathways: retrieval of contents from early endosomes or LEs to the TGN, and formation of the vesicles derived from the TGN donor membranes.

As the contents could be retrieved from either EEs or LEs, they were studied separately. First, DsRed-Rab22 and DsRed-Rab31 were used as markers for the vesicles that transport from EEs to the TGN. MCF-7 cells transfected with Rab22 and 31 were further incubated with Coumarin-6-labeled NPs at 37 °C for 3 h. The merged confocal microscopic images produced from Rab-labeled vesicles (DsRed-Rab22 and -31 positive) and Coumarin-6-positive vesicles, as shown in Figure S3C,D, implied the interaction between EEs and TGN. Then, experiments on DsRed-Rab9 cells showed that DsRed-Rab9, 22, and 31 positive vesicles merged with Coumarin-6-positive vesicles. In line with this model, NPs were transmitted from LEs to the TGN. It is clearly established that the TGN and Golgi body could receive NPs from EEs and LEs, Rab8- and 10-positive GLUT4 transport vesicles paths are employed to move NPs (Figure S3E).

### Autophagy pathways of NPs

Autophagy is a highly regulated process for eliminating a variety of intracellular materials, ranging from proteins to organelles, via lysosomal mechanisms^30^. Autophagy is a double-edged sword^31^. When tumors occur, cancer cells confer stress tolerance to stressors use autophagy, thereby maintaining tumor cell survival^30^. Autophagy is also activated when chemotherapeutic drugs invade^32^. When autophagy is induced, LC3-I is decreased, its C-terminal end is cut and LC3-II is generated from its precursor form^33^. LC3-I to LC3-II conversion is highly associated with the level of autophagosome formation, and the amount of LC3-II is an established marker of autophagy activity^11^.

**Figure 4:**
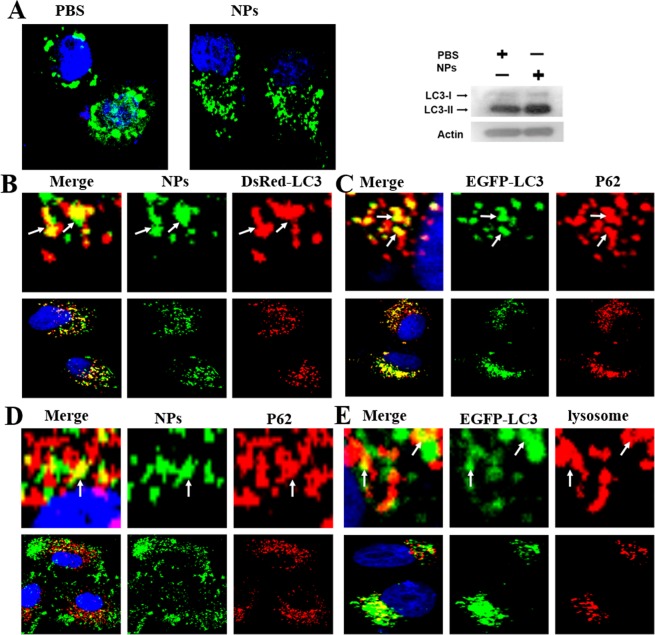
Confocal microscopy studies autophagy induced by NPs. (A) EGFP-LC3 cells were cultured with 1 mg/mL Coumarin-6-labeled NPs for 3 h. Western blotting of the cells cultured in (**A**). (**B**) DsRed-LC3 cells were cultured with 1 mg/mL Coumarin-6-labeled NPs for 3 h. (**C**) MCF-7 cells were cultured with 1 mg/mL NPs for 3 h, and P62 was detected with a primary antibody against P62. (**D**) The autophagosomes fused with lysosomes (arrows). (**E**) EGFP-LC3 cells were cultured with 1 mg/mL NPs for 3 h and Lyso-Tracker Red probes for 30 min. The above images are the enlarged ones in the white collar on the underside images. Scale bars: 10 μm.

The EGFP-LC3 transfected MCF-7 cells were incubated with Coumarin-6-labeled NPs at 37 °C for 3 h. A large amount of autophagosomes appeared, and the level of LC3-II increased **(**Fig. 4A). The cells may regard the NPs as foreign invaders. To prove that NPs can induce autophagy, we used a red fluorescent probe (DsRed-LC3) to examine autophagy. The DsRed-LC3 cells were incubated with Coumarin-6-labeled NPs at 37 °C for 3 h **(**Fig. 4B). As we speculated, there were quantities of autophagosomes in cells. P62 is a marker of selective autophagy. We treated the cells with P62 and found that P62-positive vesicles could merge with Coumarin-6-positive vesicles **(**Fig. 4C,D). Therefore, NPs can induce selective autophagy. From the results above, we hypothesized that NPs was captured by P62 and transported to autophagosomes before being degraded in lysosomes **(**Fig. 4E).

### Crosstalk among endocytosis, exocytosis and autophagy

Recent studies showed that autophagy is strongly correlated with Rab7, 8, 9, 11, 20, 32 and 33, which implies a relationship between endocytosis, exocytosis, and autophagy^26^. MCF-7 cells were co-transfected with EGFP-LC3 and DsRed-Rab proteins and incubated with Coumarin-6-labeled NPs at 37 °C for 3 h. Figure S4A–D shows that EGFP-LC3-positive vesicles merged with DsRed-Rab7, 34, 23 and 18. This finding demonstrated that autophagosomes might receive the NP vesicles from endocytosis pathways. Figure S4E,F shows that EGFP-LC3 positive vesicles merged with DsRed-Rab11 and 35, suggesting that autophagosomes might receive the NP vesicles from recycling endosome pathways. As EGFP-LC3-positive vesicles merged with DsRed-Rab8- and DsRed-Rab10-positive vesicles, we can also speculate that autophagosomes might receive the NP vesicles from GLUT4 vesicles (Figure S4G,H). Thus, there exists a complex mechanism among endocytosis, exocytosis and autophagy in cells.

### 3-MA and CQ as inhibitors of autophagy

As a double-edged sword, autophagy can not only prevent the formation of cancer but also can provide the endurance of survival for the cancer cells once the tumor formed. With the development of chemistry and biology technologies, many chemotherapeutic drugs are synthesized for different cancers. However, most can induce autophagy, which decreases the effect of drugs. We designed an experiment to test this hypothesis. Paclitaxel, doxorubicin, 5-FU and cyclophosphamide are representative chemotherapeutic drugs. MCF-7 cells were cultured with EGFP-LC3, then incubated with DMSO, paclitaxel, doxorubicin, 5-FU or cyclophosphamide at 37 °C for 24 hour. As expected, a large amount of autophagosomes was induced (Figures S5A and S6A).

3-MA and CQ are two commonly used autophagy inhibitors^31^. 3-MA inhibits autophagy via the inhibition of type III phosphatidylinositol 3-kinases (PI-3K), CQ prevents autophagy by blocking autophagosome fusion and degradation^31^. MCF-7 cells were further co-cultured with the autophagy inhibitors 3-MA and CQ and the chemotherapeutic drugs. Figures S5B–D and S6B show that there were fewer autophagosomes in the 3-MA group and more autophagosomes in the CQ group compared to the control group, indicating that 3-MA prevented the formation of autophagosomes and CQ inhibited autophagosome fusion with lysosomes.

### Inhibiting autophagy blocks the degradation of NPs in auto-lysosome pathways

**Figure 5:**
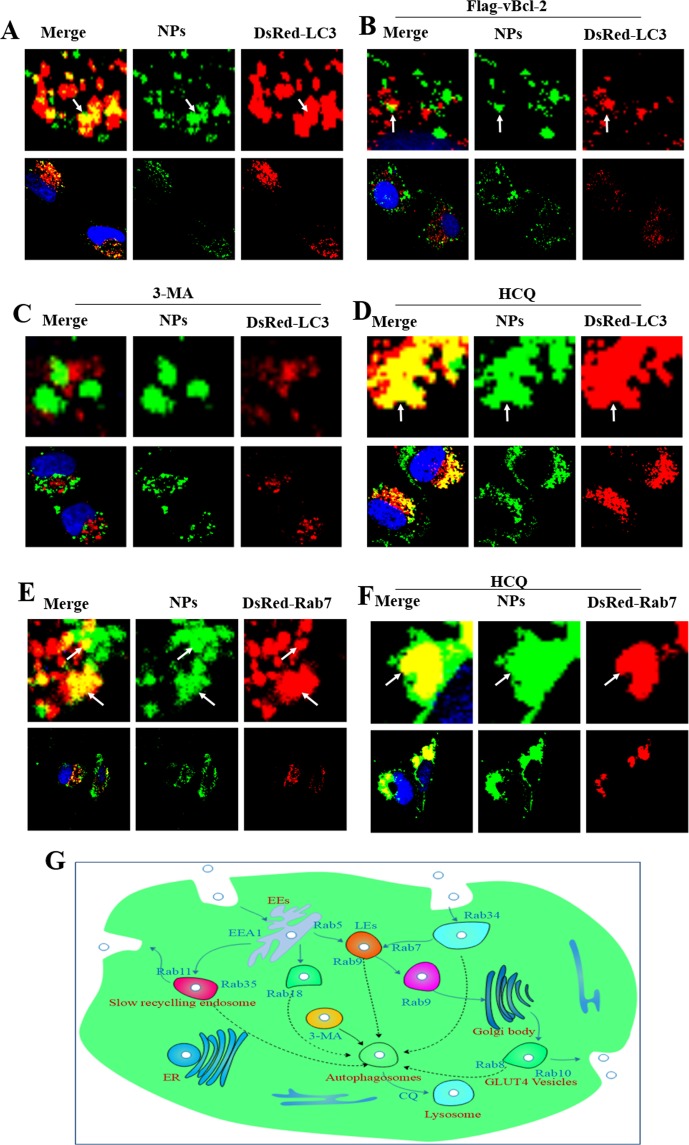
Confocal microscopy pictures of inhibiting autophagy of NPs. (**A**,**B**) DsRed-LC3 cells were cultured with 1 mg/mL Coumarin-6-labeled NPs for 3 h; DsRed-LC3 cells were co-treated with Flag-vBcl-2 and 1 mg/mL Coumarin-6-labeled NPs for 3 h. (**C**,**D**) The DsRed-LC3 cells were cultured with 10 μM 3-MA or 30 μM CQ. (**E**,**F**) DsRed-Rab7 cells were cultured with 1 mg/mL Coumarin-6-labeled NPs for 3 h; the DsRed-Rab7 cells were cultured with 30 μM CQ. (**G**) Schematic diagram of NP degradation pathways in the intracellular network. The above images are the enlarged ones in the white collar on the underside images.

Because NPs can induce autophagy and nanoparticle degradation in lysosomes, autophagy inhibitors should block the degradation of NPs through the auto-lysosome pathway. A comparative study of autophagy inhibition by 3-MA and CQ was performed. The DsRed-LC3 transfected MCF-7 cells were incubated with Coumarin-6-labeled NPs and 3-MA at 37 °C for 3 h. DsRed-LC3-positive vesicles did not merge with Coumarin -6-positive vesicles (Fig. 5A–C). Therefore, 3-MA possessed the function of inhibiting autophagy induced by NPs. In the same way, we found Coumarin-6-positive vesicles aggregated in LEs (Fig. 5D). CQ can interrupt the paths of NPs from LEs to lysosomes. We then incubated the DsRed-LC3 transfected cells with Coumarin-6-labeled NPs, CQ and DsRed-Rab7 at 37 °C for 3 hour. The control group was in the same condition but lacked CQ (Fig. 5E,F). Figure 5 shows that the result was as expected. In summary, chemical drugs such as 3-MA and CQ can not only inhibit autophagy and endocytosis pathways, but can also block NPs degraded in lysosome pathways (Fig. 5G).

### Autophagy inhibitor increased the tumor suppression effect of paclitaxel by inhibiting autophagy *in vivo*

From the previous cell experiments, we found that autophagy inhibitors such as CQ can block NPs degraded in lysosome pathways. We thus speculated that the use of autophagy inhibitors together with drugs in the nanoparticle drug delivery platform can increase the effectiveness of the chemical drugs.

**Figure 6:**
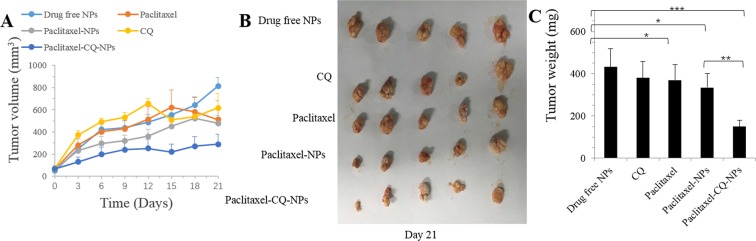
Autophagy inhibitor CQ increased the tumor suppression effect of drugs by inhibiting autophagy *in vivo*. Tumor growth curve of the SCID mice bearing MCF-7 cell xenografts after injection with drug-free NPs, paclitaxel, paclitaxel-NPs, CQ and paclitaxel-CQ-NPs. (**A**) Morphology of the tumors of each group removed from the sacrificed mice at the study end point. (**B**) Tumor weight at the study end point. (**C**) Data are shown as the means ± SD. *P < 0.05, **P < 0.01, ***P < 0.001 compared to controls.

The xenograft SCID mouse model is used to verify the curative effects. We treated the mice with drug-free NPs, paclitaxel, paclitaxel-NPs, CQ and paclitaxel-CQ-NPs every three days for seven continuous cycles. After the intraperitoneal injections, the tumor sizes were measured. The control group was injected with normal saline. Paclitaxel-NPs had an inhibitory effect on tumor growth. Paclitaxel- and CQ-NPs had similar effects on tumor growth. The paclitaxel without NPs may have been almost degraded in the lysosome. The sizes of the tumors treated with paclitaxel-CQ-NPs were markedly lower than those of the other groups, demonstrating that the cooperative use of autophagy inhibitor and chemical drugs packaged in NPs can remarkably increase the curative effect (Fig. 6).

## Conclusions

In this study, we found that NPs were internalized in cells mainly by clathrin endocytosis and caveolin endocytosis. NP movement into cells followed the classical endocytosis paths: EEs-LEs-lysosomes. We also discovered two new paths: the micropinocytosis EEs-micropinocytosis- lysosomes paths and the EEs-liposome- lysosomes paths. Endocytosis recycling and GLUT4 exocytosis vesicles were the paths through which NPs were delivered from cells. Similar to nanoparticles, NPs also induced intracellular autophagy and were then degraded via the endolysosomal pathways. Autophagy inhibitors, such as 3-MA and CQ, were able to block the endolysosomal pathways to avoid the degradation of NPs in lysosomes. The co-delivery of CQ and paclitaxel in NPs dramatically prevented the growth of tumors *in vivo*. These new intracellular network traffic mechanisms will provide new ideas for exploring the cellular behavior of nanoparticles. A new enduring and efficient drug delivery system, such as the co-delivery of an autophagy inhibitor and chemical drugs in the same nanoparticle, may be developed to help cure cancer.

## Materials and Methods

### Animals

Xenograft SCID mouse (6−8week sold) used inthe experiments were obtained from the Guang dong MedicalLaboratory Animal Center (Guangdong,China). All animal experiments followed the animal protocols approved by the Animal Care and Use Committee of Tsinghua University. Animals received care following the NSFC regulations concerning the use of experimental animals.

### Materials

PHBHHx is kindly provided by BluephaLab, China. Phosphoric acid (H_3_PO_4_), ethanol (C_2_H_5_OH), coumarin-6 and hydrochloric acid (HCl) were bought from Shenzhen Tianxiang chemical glass instrument trading company (China). Polyvinyl alcohol(PVA), dimethyl sulphoxide (DMSO), acetone (C_3_H_6_O), Chloroquine (CQ), bovine serum albumin and 3-methyladenine (3-MA) were obtained from Sigma-Aldrich (St. Louis, MO, USA). N-acryloxysuccinimide (NAS), acrylamide (AAm), N, N, N′, N′-tetramethylethylenediamine (TEMED), N, N-methylene bisacrylamide (BIS), ammonium persulphate (APS), were obtained from Aladdin Industrial Co. LTD. (Shanghai, China). Antibodies against LC3, Arf-6, RhoA, Flotillin, Caveolin, Cdc42, P62, EEA1, Clathrin, were from Cell Signaling Technology. Lyso-Tracker Red and N-(3-Aminopropyl) methacrylamide hydrochloride were from Beyotime Biotechnology (Shanghai, China) and Polymer Science, Inc., respectively.

### Preparation and characterization of PHBHHx NPs

PHBHHx nanoparticles (NPs) were formulated via a solvent displacement method^34,35^. Briefly, 5 mg PHBHHx was dissolved in 1 mL of dichloromethane, followed by intense stirring. Then, the mixture was added dropwise into 10 mL of 0.6% polyvinyl alcohol (PVA) under magnetic stirring at 800 rpm, and then stirred for 10 min. The system was left in the fume hood overnight under magnetic stirring to eliminate the dichloromethane. The NPs were collected by centrifugation at 12,000 rpm for 15 min at room temperature and washed twice. Then, the particles were dispersed in ultra-pure water and freeze-dried for at least 23 h. The freeze-dried powder was kept in the freezer until use.

The method of Coumarin-6-loaded NPs and paclitaxel-NPs was the same as described above except that 5 mg mL^−1^ and 50 µg mL^−1^ of coumarin-6 and 10 mg paclitaxel powders were added instead of using pure PHBHHx.

The drug loading content (LC) and drug encapsulation efficiency (EE) were calculated like the previously report^36^. Briefly, under vigorous vortexing, paclitaxel-NPs were dissolved in 1 mL of dichloromethane. The solution was transferred to 5 mL of mobile phase consisting of deionized water and acetonitrile (50:50,v/v). A nitrogen stream was introduced to evaporate dichloromethane for about 15 min, and then a clear solution was obtained for HPLC analysis (LC 1200, Agilent Technologies, Santa Clara, CA, USA). The measurement was performed in triplicate.

### Plasmid and transfection

DsRed-Rab5 and DsRed-Rab7 were obtained from Addgene. KSHV Flag-vBcl-2 plasmid was kindly provided by Professor Beth Levine from Department of Medicine, University of Texas. Rab family genes in the T Vector and sub-cloned into EGFP-C1 and DsRed-C1 were kindly provided by Professor Jiahuai Han’s Lab. All the plasmids were confirmed by automated DNA sequencing. And cells were transfected with the plasmids by Lipofectamine 2000 (Invitrogen).

### Characterization of NPs

The shape and structure of NPs were characterized by a scanning electron microscope (ZEISS, SUPRA-55, SAPPHIRE). Zeta potential was measured with a Malvern particle sizer Nano-ZS.

### Cell culture

The MCF-7 cells were incubate in Dulbecco’s Modified Eagle’s Medium (DMEM) supplemented with 10% Fetal Bovine Serum (FBS).

### Cellular uptake of NPs

Non-transfected or DsRed-Rab family gens were cultured with 1 mg/mL coumarin-6-label NPs at 37 °C for 3 h. Cells were incubated with Lyso-Tracker Red for 0.5 h for lysosome detection. After washing with PBS for three times, the cells were fixed with 4% paraformaldehyde for 10 min, stained with DAPI for 5 min and washed with PBS for three times. The Confocal microscopy was performed with a FLUO-VIEW laser scanning confocal microscope (Olympus, FV1000, Olympus Optical, Japan) under sequential scanning mode using a 60~100 × objective^37^.

### Autophagy assays

Cells were transfected with EGFP-LC3 under the conditions described above. Confocal microscopic images were used to calculate the EGFP-LC3 translocation. An anti-LC3 antibody was quantified by Level of LC3II protein^38^.

### Immunoblotting

Immunoblotting analysis was similar to the ref.^39^ In short, cell lysates were dissolved in 12% SDS-PAGE by immunoblotting with a LC3 antibody. An ECL detection system (Thermo Fisher Scientific, Schwerte, Germany) was used in this process.

### Immunofluorescence assay

Primary antibodies, EEA1, P62, Caveolin, Cdc42, Clathrin, LC3, Flotillin, Arf-6 were used to incubate the cells.

### Statistical methodology

All results are reported as the mean ± S.E.M. of three independent experiments. Comparisons were performed using a two-tailed paired Student’s t test. (*P < 0.05, **P < 0.01, ***P < 0.001).

## Supplementary information

supplementary info
