# Supplementary material for: Intracellular Trafficking Network and Autophagy of PHBHHx Nanoparticles and their Implications for Drug Delivery
Source: Sci Rep. 2019 Jul 3;9:9585. doi: 10.1038/s41598-019-45632-y (PMC6610140; doi:10.1038/s41598-019-45632-y)
Supplement: Supplementary file 1 — supplementary info [file 41598_2019_45632_MOESM1_ESM.docx]

Supplementary data

*of*

**Intracellular Trafficking Network and Autophagy of PHBHHx Nanoparticles and their Implications for Drug Delivery**

Xiangyu Sun^1,2,#^, Cheng Cheng^3,#^, Jinxie Zhang^1,4,#^, Xing Jin^3,^*, Shuqing Sun^1,2,^*, Lin Mei^5^, Laiqiang Huang^1,4^

^1^ Graduate School at Shenzhen, Tsinghua University, Shenzhen 518055, China

^2^ Department of Physics, Tsinghua University, Beijing 100084, China

^3^ College of chemistry and pharmaceutical engineering, Jilin Institute of Chemical Technology, Jilin, China, 132022

^4^ School of Life Sciences, Tsinghua University, Beijing 100084, China.

^5^ School of Pharmaceutical Sciences (Shenzhen), Sun Yat-sen University, Guangzhou 510275, China

# These authors contributed equally to this work.

* Corresponding authors.

Shuqing Sun: Tel/Fax: +86 075526036026, Email: sun.shuqing@sz.tsinghua.edu.cn; Xin Jin: Email: jinxing70@163.com


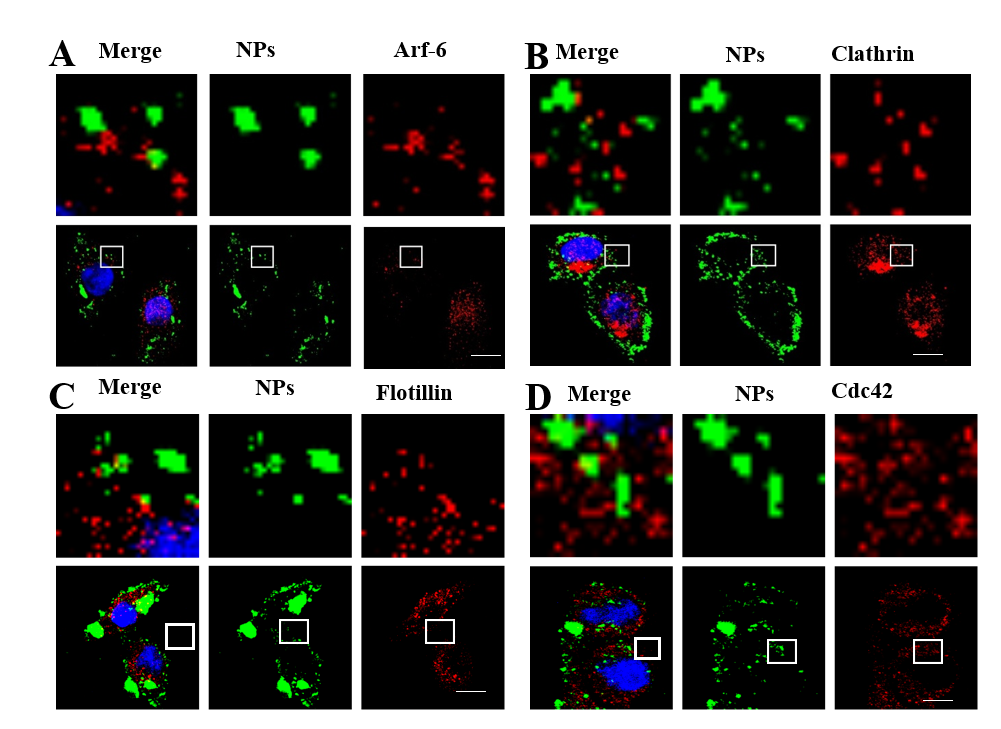


**Figure S1.** NPs cannot enter cells through Arf-6, Clathrin, Flotillin, Cdc42 endocytosis. Confocal images of MCF-7 cells, which were treated with 1 mg/mL coumarin 6-labeled NPs for 3 h. Arf-6, Clathrin, Flotillin, Cdc42 were detected with respective specific primary antibodies. The above images are the enlarged ones in the white collar on the underside images. Scale bars: 10 μm.


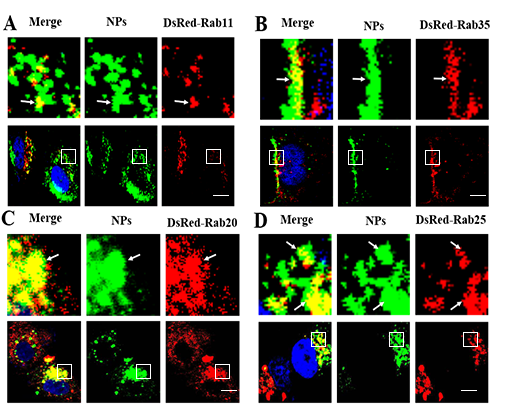


**Figure** **S2**. Confocal microscopy studies of the recycling endosome paths. (A, B) DsRed-Rab11, DsRed-Rab35 transfected MCF-7 cells were treated with 1 mg/mL Coumarin-6-labeled NPs for 3 h; (C, D) DsRed-Rab20, DsRed-Rab25 transfected MCF-7 cells were treated with 1 mg/mL Coumarin-6-labeled NPs for 3 h. The above images are the enlarged ones in the white collar on the underside images. Scale bars: 10 μm.

**
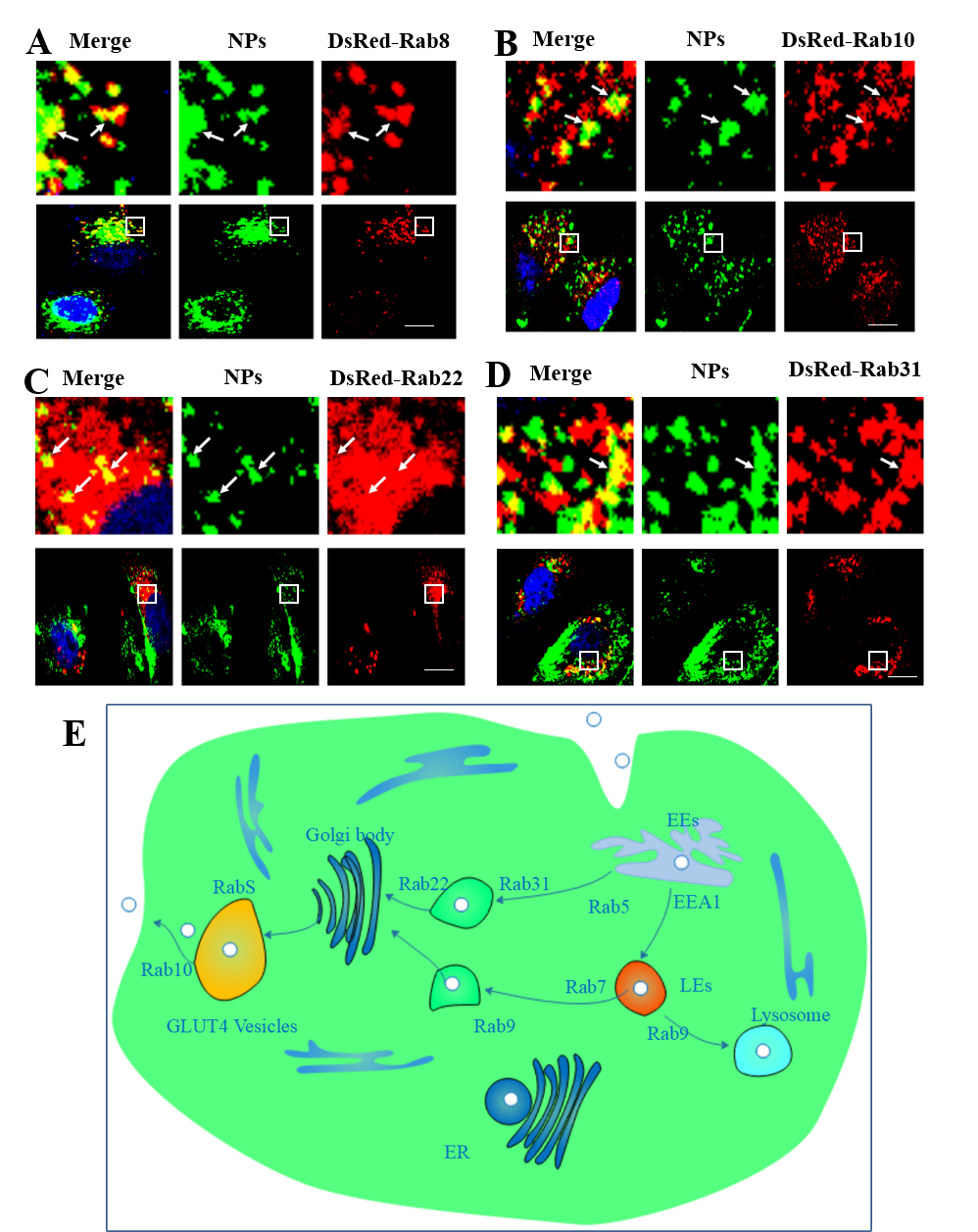
**

**Figure** **S3**. Confocal microscopy studies of the exocytosis pathways. (A, B) DsRed-Rab8 and 10 co-transfected cells were cultured with 1 mg/mL Coumarin-6-labeled NPs for 3 h. (C, D) DsRed-Rab22 and 31 co-transfected cells were cultured with 1 mg/mL Coumarin-6-labeled NPs for 3 h. (E) Schematic representation of Rab8- and Rab10-positive GLUT4 transport vesicle paths. The above images are the enlarged ones in the white collar on the underside images. Scale bars: 10 μm.


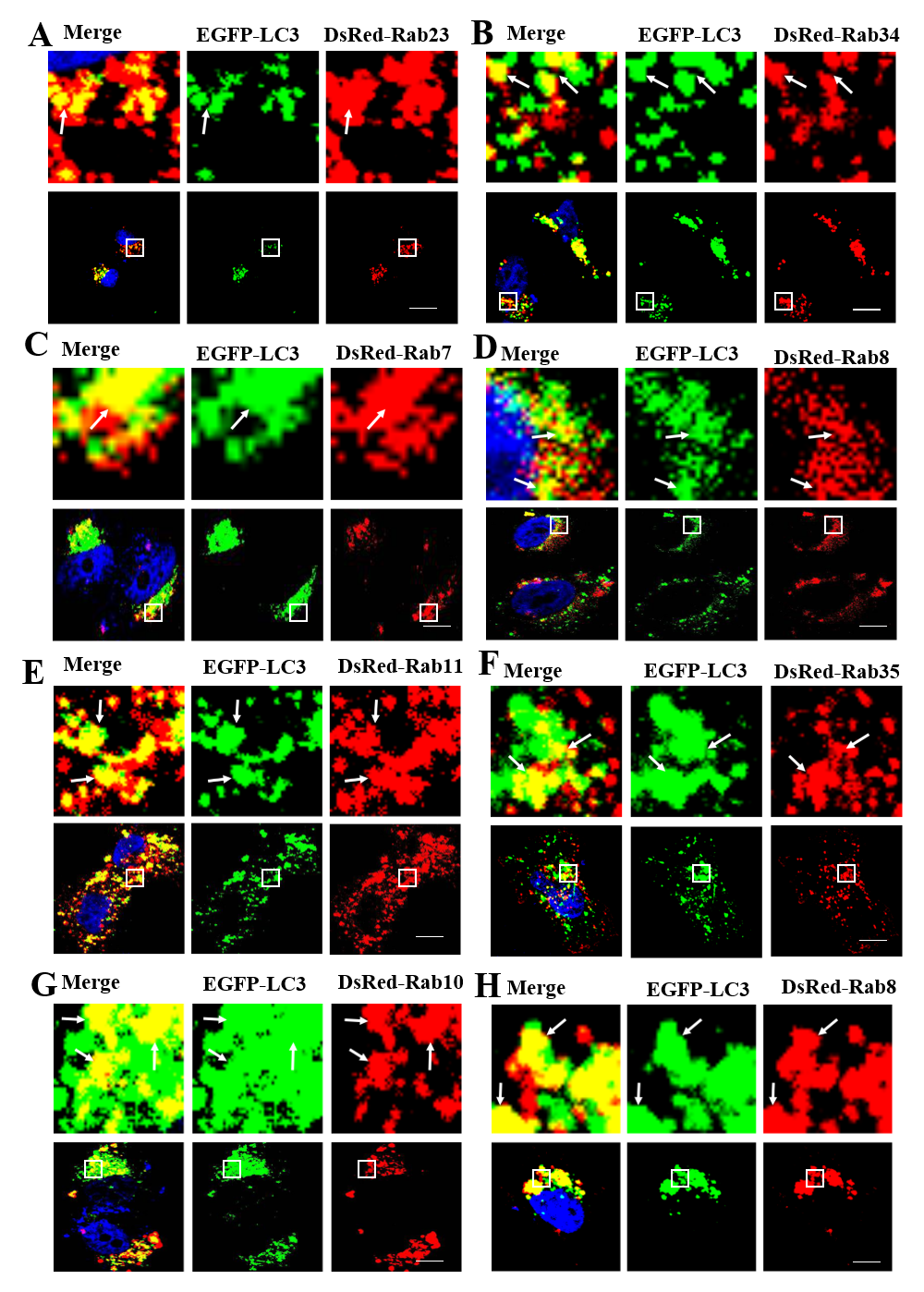


**Figure** **S4**. Confocal microscopy pictures of crosslink among endocytosis, exocytosis and autophagy. (A-H) EGFP-LC3 cells were co-transfected with DsRed-Rab23, 34, 7, 18, 11, 35, 8 and 10, respectively. The above images are the enlarged ones in the white collar on the underside images. Scale bars: 10 μm.


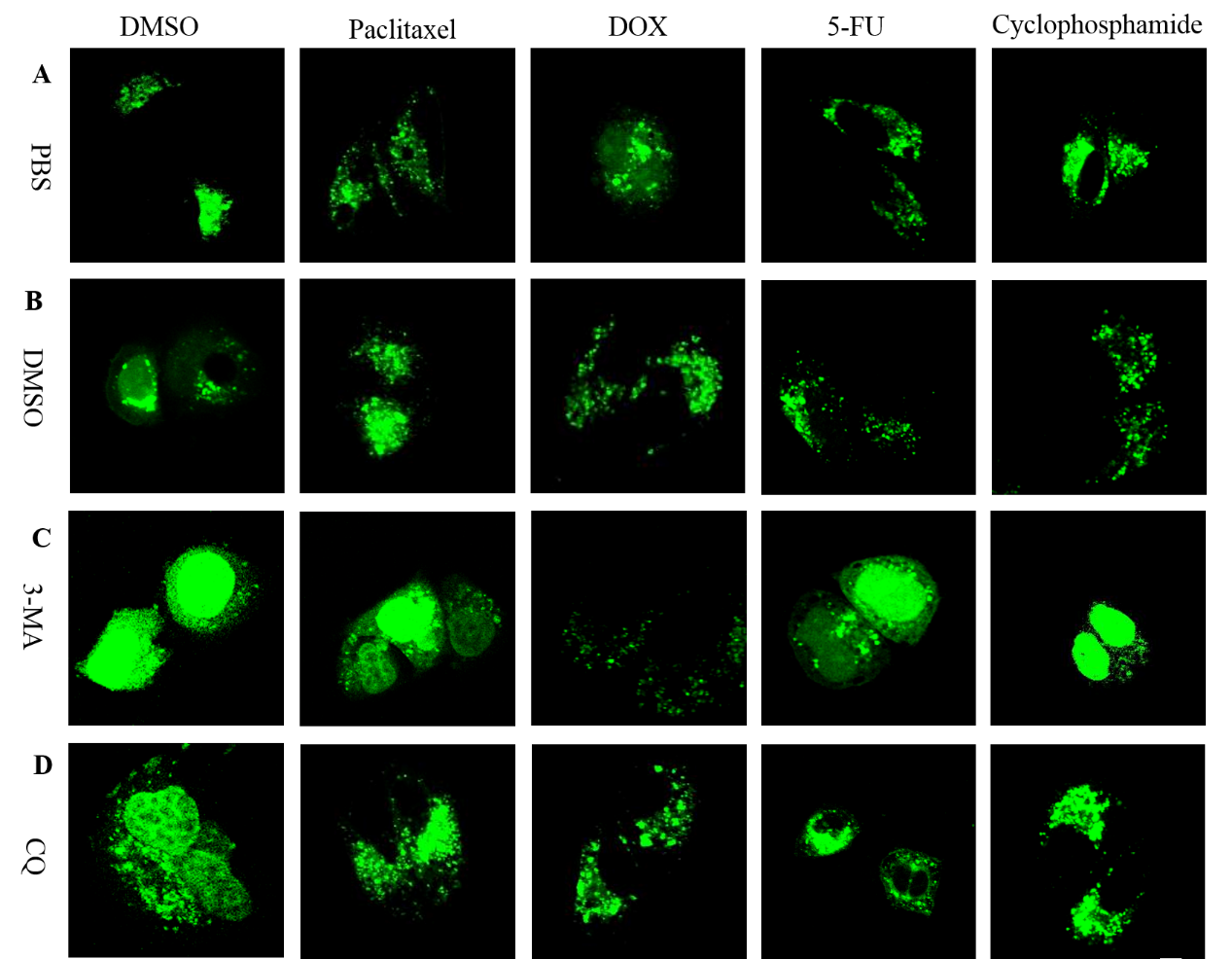


**Figure S5**. 3-MA and CQ inhibit autophagy which induced by chemotherapeutic drug. (A) The EGFP-LC3 cells were cultured with 1 mM paclitaxel, 10 mM DOX, 10 mM 5-FU, or 10 mM cyclophosphamide for 24 h. (B, C and D) The EGFP-LC3 cells were treated with 10 mM 3-MA or 30 mM CQ with 1 mM paclitaxel, 10 mM DOX, 10 mM 5-FU, or 10 mM cyclophosphamide for 24 h. Scale bars: 10 mm.


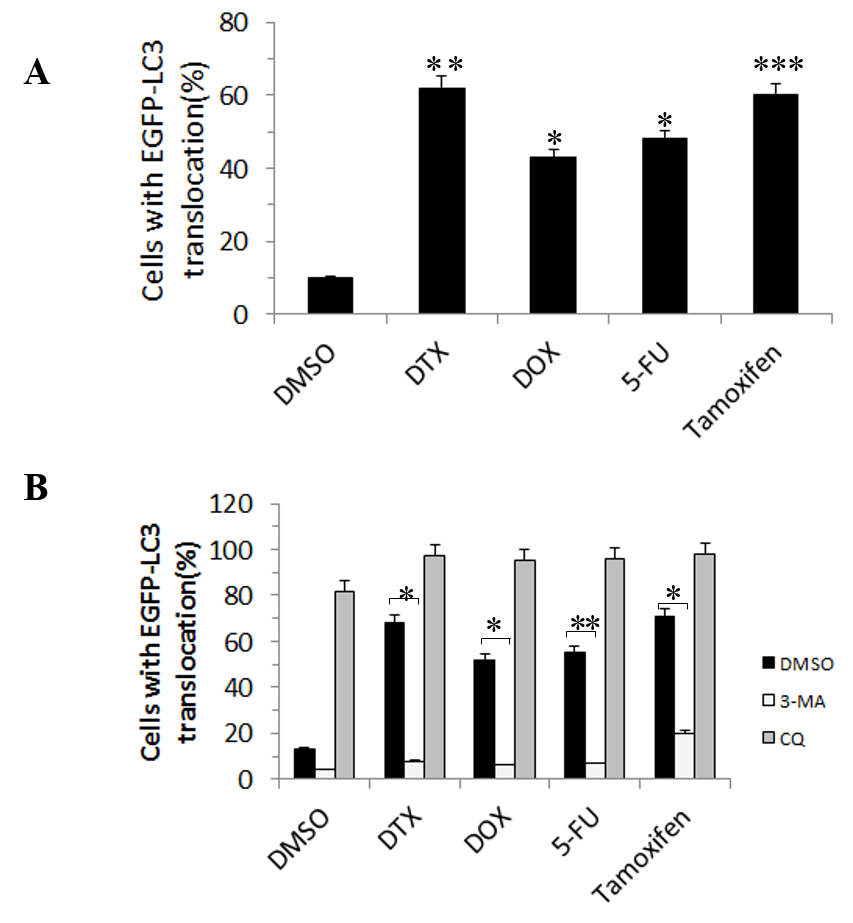


**Figure** **S6**. The level of cells with EGFP-LC3 translation. respectively. Data are shown as the means ± SD. *P < 0.05, **P < 0.01, ***P < 0.001 compared to controls.
